# Intrinsic timescales across the basal ganglia

**DOI:** 10.1038/s41598-021-00512-2

**Published:** 2021-11-01

**Authors:** Simon Nougaret, Valeria Fascianelli, Sabrina Ravel, Aldo Genovesio

**Affiliations:** 1 Institut de Neurosciences de la Timone, UMR7289, Centre National de la Recherche Scientifique and Aix-Marseille Université, Marseille, France; 2 Department of Physiology and Pharmacology, Sapienza University of Rome, Rome, Italy; 3 PhD Program in Behavioral Neuroscience, Sapienza University of Rome, 00185 Rome, Italy

**Keywords:** Cognitive neuroscience, Neural circuits

## Abstract

Recent studies have shown that temporal stability of the neuronal activity over time can be estimated by the structure of the spike-count autocorrelation of neuronal populations. This estimation, called the intrinsic timescale, has been computed for several cortical areas and can be used to propose a cortical hierarchy reflecting a scale of temporal receptive windows between areas. In this study, we performed an autocorrelation analysis on neuronal populations of three basal ganglia (BG) nuclei, including the striatum and the subthalamic nucleus (STN), the input structures of the BG, and the external globus pallidus (GPe). The analysis was performed during the baseline period of a motivational visuomotor task in which monkeys had to apply different amounts of force to receive different amounts of reward. We found that the striatum and the STN have longer intrinsic timescales than the GPe. Moreover, our results allow for the placement of these subcortical structures within the already-defined scale of cortical temporal receptive windows. Estimates of intrinsic timescales are important in adding further constraints in the development of computational models of the complex dynamics among these nuclei and throughout cortico-BG-thalamo-cortical loops.

## Introduction

Organization of the brain has been described following different principles. For example, areas can be organized based on the laminar pattern of origins and terminations of cortico-cortical projections^1,2^ or based on topological projection sequences^3^. Following a proposed anatomical hierarchy of the visual, somatosensory, and motor cortices^1^, and considering the laminar structure of the prefrontal cortico-cortical projections, prefrontal areas are at the top of this hierarchy^4^. Interestingly, this anatomical hierarchy is mirrored by the intrinsic fluctuations in spiking activity across these areas at rest^4,5^. Computed from their spike-count autocorrelation, these intrinsic timescales are considered to be a measure of temporal stability of the neuronal activity. By comparing different cortical areas, past studies^4,6^ have shown that prefrontal areas have the longest timescales, the posterior parietal and the dorsal premotor cortex have intermediate timescales, and the primary somatosensory cortex has the shortest timescale. The proposed cortical hierarchy^4,7^ is intended to reflect a scale detailing temporal receptive windows with higher-level areas with the longest timescales representing the progressive accumulation of neuronal inputs and supporting high-level cognitive decision-making processes.

All cortical areas except for the primary visual and auditory areas project to the basal ganglia (BG)^8^, which serve as the substrate of several cognitive processes such as context- and value-based decision-making, reinforcement learning, inhibition control, and working memory. Here, we used a similar method to Murray and colleagues^4^ to analyze the intrinsic timescales of neuronal populations in the striatum (phasically active neurons or PANs, putative projection neurons), the subthalamic nucleus (STN), and the external globus pallidus (GPe) of macaque monkeys during the baseline period of a visuomotor task^9,10^. We found that the input structures of the BG, the striatum and the STN, exhibited longer timescales than the GPe. Describing the differences between the timescales of these populations can help lead to a better understanding of the functional specialization of these structures and validate computational models of action selection.

## Results

We analyzed neuronal activity during the baseline period of a visuomotor task as described by Nougaret and Ravel^9,10^. This period was suitable for our analysis that aimed to capture the neuronal activity not related to the neural processes engaged during the task. We computed the spike-count autocorrelation structure for each neuron as a function of time lag, and estimated its decay constant (intrinsic timescale τ) with an exponential fit. We assigned the intrinsic timescale to the whole neuronal population and to single neurons as described in “Material and methods”. The database we analyzed consisted of 78 neurons recorded in the STN (30 and 48 from monkey M and monkey Y, respectively); 158 PANs recorded in the striatum, presumed to be medium spiny projection neurons^11^ (96 and 62 from monkey M and monkey Y, respectively); and 92 irregular neurons, corresponding to high-frequency discharge neurons (HFD)^12^, from the GPe (41 and 51 from monkey M and monkey Y, respectively). Only neurons from the GPe were analyzed in a previous study^10^. Localizations of PANs in the striatum and STN neurons were assessed as in previous studies^9,10^ using MRI scans with electrodes for locating trajectories, from which the neurons were recorded.

### Intrinsic timescales of STN, PANs, and GPe

To assess the spike-count autocorrelation values as a function of time lags, we required a non-zero mean activity for each neuron in each 50 ms bin during the baseline period. In particular, 77/78 neurons in the STN, 103/158 PANs in the striatum, and 92/92 neurons in the GPe fulfilled this first requirement (see “Material and methods”). We further reduced the neuronal sample by requiring each single timescale larger than zero, an R^2^ obtained from the fit procedure larger than 50%, and by an accurate eye inspection as described in “Material and methods”. After applying this selection criteria, we reduced our neural population to 14/78, 39/158, and 71/92 neurons in STN, striatum, and GPe, respectively. The electrophysiological characteristics of each neuronal population are summarized in Table 1.

**Table 1:** Electrophysiological characteristics of the selected neuronal populations.

	Number of neurons	Number of trials	Firing rate (spike.s^−1^)	Inter-spike interval (ms)	CV of the inter-spike interval
STN	14	81.0 ± 18.0	19.5 ± 14.2	60.7 ± 32.5	0.83 ± 0.24
Striatum (PANs)	39	85.4 ± 11.5	5.0 ± 3.9	93.1 ± 26.6	1.22 ± 0.18
GPe	71	78.3 ± 9.7	60.0 ± 20.9	18.4 ± 7.3	1.10 ± 0.36

Each row represents a structure of the basal ganglia (STN for Subthalamic Nucleus, PANs for the Phasically Active Neurons of the Striatum and GPe for the external segment of the globus pallidus). The columns show the number of neurons selected for the analysis, the number of trials during which they were recorded and the mean and standard deviation of their electrophysiological characteristics. The coefficient of variation of the inter-spike interval (ISI) was computed by dividing the standard deviation of the ISI by the mean of the ISI.

**Figure 1:**
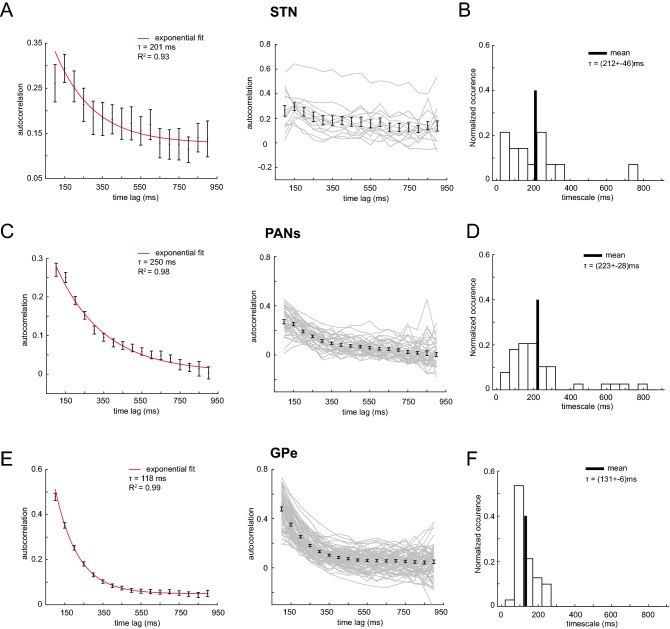
Mean autocorrelation values and single timescale distribution. (**A**) Left Panel: mean autocorrelation averaged across all neurons (n = 14) recorded in the subthalamic nucleus (STN) using 50 ms time bins in a 900 ms time window during the baseline period (mean ± SEM). The solid red line is the exponential fit. The autocorrelation at 50 ms has been excluded from the fit procedure. The intrinsic timescale τ is shown in the top right corner, with the R^2^ value as a goodness of fit estimator. Right panel: single neuron autocorrelation structure (grey lines) with the mean and standard error bar for each time lag (black bars). (**B**) Single neuron timescale distribution for STN (n = 14) computed in the same baseline period as in the population timescale shown on the left panel. The solid line is the mean. The mean of the timescale distribution is shown in the top right corner with the standard error of the mean. The median of the distribution is 178 ms, while the mode gets two values, 50 ms and 200 ms. (**C**) Left Panel: mean autocorrelation averaged across neurons (n = 39) recorded in phasically active neurons (PANs) of the striatum in the same baseline period as (**A**). The autocorrelation value at 50 ms has been excluded from the fit procedure as in A). Right panel: same as in (**A**) right panel. (**D**) Single neuron timescale distribution for PANs (n = 39). The median of the distribution is 181 ms, while the mode gets two values, 50 ms and 150 ms. (**E**) Left Panel: mean autocorrelation averaged across neurons (n = 71) recorded in the external globus pallidus (GPe) in the same baseline period as (**A**) and (**C**). The autocorrelation values at all time lags have been included in the fit procedure. Right panel: same as in (**A**). (**F**) Single neuron timescale distribution for GPe neurons (n = 71). The median and mode of the distribution are 115 ms, and 100 ms, respectively.

Figure 1 (left) shows the autocorrelation values as a function of time lags averaged across neurons for each brain structure, with the exponential fit superimposed along with the estimated timescale τ and its interval at 95% confidence level. In particular, the GPe showed a shorter timescale (118 ms [113–124]) than both the striatum (250 ms [198–300]) and the STN (201 ms [112–289]). We noticed that the timescale estimate of STN showed a broad confidence interval, and this is likely the effect of the small size of the neuronal sample (14 neurons). To overcome this issue and to assess whether GPe timescale was significantly smaller than the striatum and the STN timescales, we compared the sample mean of the single timescale distributions (see “Material and methods”). The mean (± standard error of the mean, sem) of the single timescale distributions is 212 ± 46 ms, 223 ± 28 ms, and 131 ± 6 ms for STN, striatum, and GPe, respectively (see Figs. 1B, D, F). We computed the absolute value of the difference between GPe-striatum timescales (91 ± 29 ms), and GPe-STN timescales (80 ± 47 ms), and both differences are not compatible with zero within the error, meaning that GPe showed a significantly shorter timescale than both the striatum and STN (see “Material and methods” for quantification of the difference between the mean of the timescales’ distributions based on the general propagation of the error rule). Moreover, the difference between STN-striatum timescales (11 ± 54 ms) is compatible with zero within the error, meaning that the intrinsic timescale of the STN is compatible with the timescale of the striatum.

Comparing the timescales of the BG structures, it is worth noticing that the standard error of the GPe mean timescale is smaller than the other two BG structures. This result might be explained by the lower heterogeneity in the autocorrelation structures of the GPe single neurons compared to that of the neurons of the other two BG structures. We found a significant difference in the heterogeneity of the timescale values of GPe neurons and PANs (two-sample *F* test for equal variances, p = 9e−18), and GPe and STN neurons (two-sample *F*-test for equal variances, p = 3e−12). No significant difference in heterogeneity was found between STN neurons and PANs timescale distributions (two-sample *F* test for equal variances, p = 0.9). In addition, to investigate the degree of heterogeneity in the single timescale distributions for each BG structure, we computed the coefficient of variation (CV) of the timescale distribution using formula (7) in “Material and methods”. We found a higher degree of heterogeneity in STN neurons (CV = 15%) and PANs (CV = 12%) τ distributions than in the GPe neurons τ distribution (CV = 7%), in line with the previous individual timescale distribution parameters, i.e. mean and standard error of the mean (sem), that were smaller for GPe neurons than for STN neurons and PANs.

## Discussion

To our knowledge, our study is the first to report estimations of intrinsic timescales of neuronal populations at the subcortical level, and reveals timescale differences between the input structures of the BG, the striatum and the STN, and the GPe. Earlier studies have used the autocorrelation function to understand the firing pattern properties of single cells within the BG^13–15^ and midbrain dopaminergic neurons^16^. Specifically, autocorrelograms of single cells have been used to classify these cells into different subpopulations, for example into GPe neurons^17^ or different types of dopaminergic neurons^16^, and to assess firing rate rhythmicity of single neurons from BG nuclei in healthy and diseased conditions^13,14,18,19^. In this study, we used the autocorrelation function to characterize the properties of neuronal populations in the BG and place them in the context of already-known intrinsic timescales throughout the cortex.

**Figure 2:**
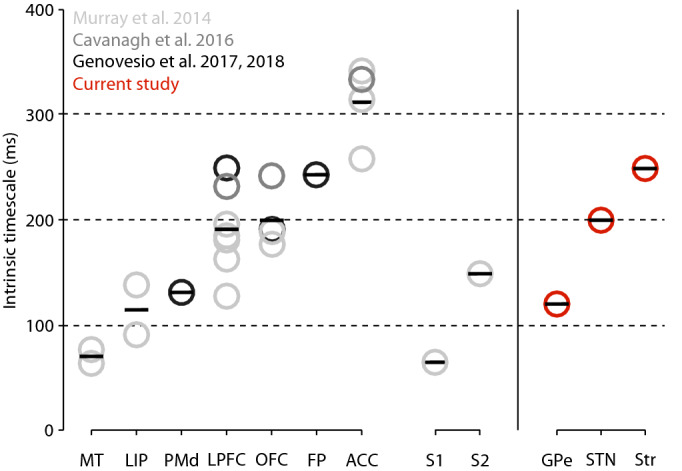
Hierarchical organization of intrinsic timescales of cortical and subcortical structures. Left Panel: Intrinsic timescales of nine cortical areas reported by^4^ in light gray, by^20^ in medium gray, and by^6,21^ in dark gray. The seven areas on the left (MT, LIP, PMd, LPFC, OFC, FP, and ACC) are part of the visual and prefrontal cortices. The two areas on the right are part of the somatosensory cortex (S1, S2). Each circle represents the average τ for each cortical area reported in each study. Each bar represents the average τ among the studies. Right Panel: Same representation for the three subcortical structures (GPe, STN, and striatum) analyzed in the present study. ACC, anterior cingulate cortex; FP, frontopolar cortex; GPe, external globus pallidus; LIP, lateral intraparietal cortex; LPFC, lateral prefrontal cortex; MT, medio-temporal area (of visual cortex); OFC, orbitofrontal cortex; PMd, dorsal premotor cortex; S1, primary somatosensory cortex; S2, secondary somatosensory cortex; STN, subthalamic nucleus.

We found that the striatum and the STN exhibited longer timescales (250 and 201 ms respectively) compared to the GPe (118 ms). A cortical hierarchy has already been described^4^ based on values from seven cortical areas (Fig. 2, light gray circles), placing the prefrontal areas, anterior cingulate cortex (ACC; average value = 303 ms), orbitofrontal cortex (OFC; average value = 182 ms), and lateral prefrontal cortex (LPFC; average value = 166 ms) at the top of this hierarchy with the longest timescale values. The same study then reported intermediate timescales in the lateral intraparietal cortex (LIP; average value = 114.5 ms) and the secondary somatosensory cortex (S2), and the shortest timescales in the medio-temporal area (MT) of the visual cortex and the primary somatosensory cortex (S1). Other studies (^20^, medium gray circles;^21^, dark gray circles) later confirmed the previous results overall, reporting comparable LPFC (231/248 ms), OFC (241/190 ms), and ACC (332 ms) timescale values. Genovesio and colleagues^6,21^ then extended the hierarchy previously described by assigning intrinsic timescales to the frontopolar cortex (PFp; 242 ms) and the dorsal premotor cortex (PMd; 131 ms).

The striatum and STN intrinsic timescales reported here place these structures on a comparable level as the timescales assigned to prefrontal areas. Importantly, in both of these BG structures recordings were mainly from their associative parts, known for receiving their major inputs from the prefrontal cortex^22–25^. In contrast, the GPe exhibited an intrinsic timescale of only 118 ms, which places it at the same intermediate level as the PMd, LIP, and S2. One possibility is that this lower timescale could reflect lower temporal information integration ability of the GPe among BG structures. Our study shows a gradient of receptive time windows within BG circuitry (Fig. 2, red circles). As suggested for areas of the visual system^5^, the differences found among the BG nuclei could reflect a broader window of temporal information storage in the striatum and the STN compared to in the GPe. Indeed, intrinsic timescales could be described as “a temporal counterpart of the spatial hierarchy”^7^. Previous studies^4,26^ shed light on the existence of a parallel between the anatomical hierarchy of cortical areas and their intrinsic timescales. Drawing a parallel with the cortex, the interpretation of a functional hierarchy based on timescales described for cortical areas might apply to the BG. This would suggest a greater need for information accumulation within the BG input nuclei rather than in the GPe. The convergence of information from multiple cortical areas could explain the necessity for longer timescales in both input structures because of the need of striatal projection neurons and subthalamic neurons to gate and maintain information from prefrontal neurons^27,28^.

A large body of work in primate neurophysiology has shown that, at the neural level, choosing corresponds to crossing a firing rate threshold in the cortex. Depending on the environment and on the decision being made, the threshold level can be regulated. A computational study^29^ implemented a biophysically-based network model of decision thresholds of the cortical-BG-superior colliculus (SC) pathway. After comparing the different nodes of these networks, the authors concluded that the all-or-none activity of SC neurons is triggered by a threshold crossed by cortical neurons that can be optimally tuned by the strength of cortico-striatal synapses. Indeed, through this pathway, the output structures of the BG inhibit cortex and SC activity to preclude inappropriate motor outputs^30^. Some authors^31^ have hypothesized that “the BG may convert cortical representations of sensory evidence into evaluative quantities,” allowing generation and adjustment of decisions. They suggest that the BG can modify the decision rules by modifying the decision threshold, but also modify the “value of a developing decision variable.” Different BG models hypothesize that the main role of the BG nuclei is to act as a central selection device^32^ that examines each action requested based on its urgency and salience^33^ and that, with their unique anatomical properties, allows the allocation of motor resources to the appropriate actions. The striatum and the STN have distinct roles in these processes. According to Frank and colleagues, the former has a crucial role in gating sensory input for updating working memory in the prefrontal cortex and then in maintaining it, preventing the influence of distracting information^27^, especially when adaptive gating is necessary for the processing of multiple goal demands. The STN is supposed, particularly during high-conflict decisions, to reduce premature responses and to refine the selection process that takes place via cortico-striatal pathways^28,34,35^. This special role could be conferred by the direct afferences it receives from prefrontal areas^24,25^ and the top-down signal it sends to the striatum through the GPe. A computational study suggests that during a countermanding task, after the appearance of an infrequent stop signal, STN acts as a brake to cancel forthcoming actions and keeps accumulating the evidence of the stop signal over time along the decision process^36^. As a consequence, the STN is indirectly involved in higher cognitive functions that require long timescales as for prefrontal areas^26,37^.On the other hand, models of BG action selection assign another role to GPe neurons. Gurney and colleagues^33,38^ have proposed that the GPe, mainly through its massive projections into the STN, automatically limits the activity of BG output structures and allows the network to make a selection. The control that the GPe exerts on other BG nuclei is supported by in vitro electrophysiological studies showing that the firing rate of GPe neurons can be approximated by a linear function of the injected current^39^. In contrast, the firing of the STN neurons fits better with an exponential function of their inputs^33^. This understanding is also consistent with in vivo electrophysiological studies in primates that report independent encoding of task variables by GPe neurons^10,40^, suggesting more a parallel processing of information by GPe neurons rather than an integration of different variables^10^. Taken together, these results support a lack of integration of information at GPe level and are in line with the intermediate intrinsic timescale exhibited by the GPe in the present study. However, a recent computational study^26^ suggests caution around assigning timescales to brain areas too rigidly, because processing different sensory inputs may lead to different timescales based on their model. Moreover, the three BG nuclei studied here exhibited different degrees of heterogeneity in their single unit timescales (Fig. 1, left panel). In particular, the input structures displayed a higher degree of heterogeneity than the GPe. Some studies have shown that within each cortical area, the individual intrinsic timescale computed during a baseline period predicted the strength of response modulation during following task periods in the LIP^41^, PMd^6^, and dlPFC^21^, suggesting that neurons with longer timescales are more involved in the encoding of task-related information. We could hypothesize that the high degree of heterogeneity found in the input structures of the BG could serve to support the heterogeneity of information that these structures have to process with different temporal integration requirements, although further study is necessary to reach conclusions about these functions.

Our study is the first to quantify intrinsic timescales of BG nuclei, and some limitations should be considered. First, our datasets are relatively small compared to others used for the cortex and have high variability at the population level. This is mainly true for the input structures, which showed a higher degree of heterogeneity at the single-cell timescale level. Moreover, our selection criterions excluded a large number of cells, especially in the input structures, suggesting that the decay constant of only a subpopulation of PANs and STN neurons can be well estimated with an exponential fit. Second, the BG nuclei are known to be partially specialized in sensorimotor, associative, and limbic territories, and our datasets cover mainly the associative and the limbic parts of these structures. For future studies, it remains to be investigated how our BG nuclei timescale estimates could be generalized at the whole-structure level, and whether our estimates are consistent with the timescales of other BG nuclei not studied here. Third, we performed our analysis during the baseline period as in^4^. It offered the possibility to compare the timescales values of subcortical and cortical structure but did not fully represent the neuronal activity at rest. It is also important to investigate the relationships within each structure at the single-cell level between timescales and show persistent representations of task-relevant signals, as has been done in the cortex^6,20,21,39^. We believe that notwithstanding these limitations, the timescales reported here could be useful as a first approximation for the validation of computational models of action selection based on evidence accumulated through cortico-striatal/subthalamic synapses and architecture of cortico-BG-cortical loops, as has been done for the cortex^26^.

## Material and methods

### Resource availability

Further information and requests for resources and reagents should be directed and will be fulfilled by Simon Nougaret (simon.nougaret@univ-amu.fr).

The datasets and code supporting the current study have not been deposited in a public repository but are available from the corresponding author on request.

### Use of experimental animals

All experimental procedures were conducted in conformity with the French laws on animal experimentation, with the European directive 2010/63/UE on the protection of animals used for scientific purpose and with the ARRIVE guidelines. All experimental protocols were reviewed and approved by the local ethical committee of the Institut de Neurosciences de la Timone.

### Experimental model and subject details

Two male rhesus monkeys (*Macaca mulatta*) were used in this study. Relevant details about the animals have already been reported in^9,10^.

### Dataset

The experimental details of the datasets used in the current study have already been reported^9,10^. The single-unit activity of two male rhesus monkeys (*Macaca mulatta*), recorded from three populations of neurons within the (BG), were analyzed during a foreperiod, considered as a baseline period in which no cognitive process was engaged. During this 1 s period, both monkeys needed to maintain a basal pressing force on a lever and wait for the presentation of a pair of visual stimuli indicating the amount of force needed and the amount of reward to be expected at the end of the trial. The data set consisted of 1) a population of 158 putative projection neurons recorded in the striatum, also called phasically active neurons (PANs); 2) a population of 78 neurons from the subthalamic nucleus (STN); and 3) a population of 92 irregular neurons from the external globus pallidus (GPe).

### Quantification and statistical analysis

#### Neuronal sample selection and population timescale analysis

We analyzed the recorded activity of neurons in the STN, GPe, and striatum (PANs) from 100 ms after the beginning of the trial until the end of the baseline period for a total of 900 ms of baseline activity. The same baseline period had already been chosen^9^ for electrophysiological analysis of neurons recorded for the same task. We included only correct trials in the following analyses. For each structure, we selected neurons satisfying the following criterium:

1 $${1}{\text{.}}\quad {\text{each}}\,{5}0{\text{ - ms}}\,{\text{time}}\,{\text{bin}}\,{\text{in}}\,{\text{the}}\,{\text{baseline}}\,{\text{period}}\,{\text{with}}\,{\text{non - zero}}\,{\text{mean}}\,{\text{activity}}\,{\text{across}}\,{\text{trials}}.$$

We performed all analyses with MatLab (The MathWorks, Inc., Natick, MA, USA). For all the following fit procedures, we used the Trust-Region algorithm for Nonlinear Minimization problem implemented in the Optimization Toolbox in Matlab.

To assess the single neuron spike-count autocorrelation structure, we calculated the spike count during the baseline period in 50 ms time bins. It is worth noting that the results did not change within a difference of 20% of the bin length. Given a neuron, the spike-count autocorrelation value across trials between time bins *k* and *j* (*k, j* as integer numbers) at a time lag equal to |*k-j*|x *Δ* (*Δ* = 50 ms), the Pearson’s correlation coefficient *r* is defined as follows^4^:

2 $$r = \frac{Cov(N(k),N(j))}{\sqrt{Var(N(k))\times Var(N(j))} }= \frac{< (N(k) - \overline{N(k)})(N(j) -\overline{ N(j)}) >}{\sqrt{Var(N(k))\times Var(N(j))}}$$

where *N(k)* and *N(j)* are the spike counts computed in the *k* and *j* time bins, respectively, and $\overline{N\left(k\right)}$ and $\overline{N\left(j\right)}$ are the spike counts averaged across trials in *k* and *j* time bins, respectively. The covariance (*Cov*), the variance (*Var*), and the autocorrelation value *r* were computed for each possible combination of pair-bins (*k, j*). We calculated the autocorrelation values as a function of the time lags for each neuron satisfying the criterion in (1). We subsequently performed an exponential fit to each neuron’s autocorrelation as defined below^4^:

3 $$r(n\varDelta ) ={ A}\left[exp(-\frac{n\varDelta }{\tau })+{B}\right],$$

where *nΔ* indicates the time lag between the time bins *k* and *j*, with *n* =|*k*—*j* | (*n* = 1,2, …, 18); *r* is the autocorrelation value at time lag *nΔ*; A is the amplitude; τ is the decay constant of the exponential function, called intrinsic timescale; and B is the offset that mirrors the value of *r* in the limit of time lag *nΔ* → ∞ (i.e., time lag values much larger than our 900 ms baseline length). Throughout this paper, we refer to intrinsic timescale as simply timescale or τ.

We further reduced the neuronal sample by selecting those neurons satisfying both the criterion in (1) and the following requirements:

4 $$\begin{aligned} & 2.\quad \tau > { }0\,{\text{ms}}; \\ & 3.\quad {\text{R}}^{2} > 50{{\% }}, \\ \end{aligned}$$

where R^2^ is the coefficient of determination obtained by the fit. The first requirement was introduced because a negative or 0 ms *τ* value is meaningless; the second requirement of an R^2^ larger than 50% was a trade-off between the need to keep as many neurons as possible and the importance of having a good fit. We also excluded outliers, defined as neurons having an intrinsic timescale below the 5th percentile and above the 95th percentile of the *τ* distribution, from the sample. This last requirement was established due to the heterogeneity of timescale values within each brain structure and to avoid having an estimate of the mean of the intrinsic timescales biased towards the outlier values.

Moreover, we decided to discard those neurons poorly fit by the exponential function by eye inspection^20^. In more detail, we removed a neuron whether the autocorrelation values had an oscillatory behavior with increasing time lags, or the autocorrelation values showed a flat shape with increasing time lags.

In order to assess the timescale of the whole neuronal population, we first averaged the coefficient *r* defined in formula (2), at a fixed time lag, across neurons satisfying all the previous selection criteria. We subsequently fit the averaged autocorrelation values at increasing time lags with the exponential function defined in (3). The estimate of the decay constant τ was the timescale assigned to the neuronal population for each brain structure.

#### Single-neuron intrinsic timescale analysis

We moved further by characterizing the single neuron timescale distribution. For each brain structure, we computed the mean of the single timescale distribution. To compare the sample mean of the timescale distributions across brain structures, we applied the following procedure. Given 2 sample means τ1 and τ2, with 2 standard errors of the mean, sem1 and sem2, respectively, we computed the difference ∆:

5 $$\Delta = |\uptau 1-\uptau 2|.$$

In order to assess whether the difference ∆ is compatible with zero (i.e. no significant difference between the 2 mean timescales of the distributions), we quantified the error associated to ∆ as follows:

6 $$\mathrm{err}(\Delta ) = \sqrt{({\mathrm{sem}1)}^{2 }+{(\mathrm{sem}2)}^{2 }},$$

that stems from the general propagation of the error rule. If the value of ∆ is compatible with 0, within the error range, we would say that there is no significant difference between the mean of the timescales distributions.

We further investigated the degree of heterogeneity of the single timescale distribution for each brain structure. To quantify this, we used the coefficient of variation (CV), defined as follows:

7 $${\text{CV}}\, = \,{\text{sigma}}/{\text{mean}},$$

where sigma and mean are the standard deviation and mean of the timescale distribution, respectively.
